# Navigating Diagnostic Difficulties: Benign Early Repolarization/Subtle ST Elevation in a Young Patient Presenting As Myocardial Infarction

**DOI:** 10.7759/cureus.89632

**Published:** 2025-08-08

**Authors:** Abdul Samad, Harshvardhan P Thakar, Muhammad Hassan, Jasmine Kaur, Ali Tariq Hussein

**Affiliations:** 1 Acute Medicine/Emergency, Pilgrim Hospital, United Lincolnshire Hospitals NHS Trust, Boston, GBR; 2 Internal Medicine, Pilgrim Hospital, United Lincolnshire Hospitals NHS Trust, Boston, GBR; 3 Acute Medicine, Pilgrim Hospital, United Lincolnshire Hospitals NHS Trust, Boston, GBR; 4 Oncology, Pilgrim Hospital, United Lincolnshire Hospitals NHS Trust, Boston, GBR; 5 Emergency Medicine, NMC Royal Hospital, Khalifa City, ARE

**Keywords:** benign early repolarization (ber), myocardial infarction, subtle st elevation, unusual causes of persistent chest pain, young adult male

## Abstract

In adults having chest pain, ST-segment elevation, both benign and pathologic, is a common finding seen on electrocardiograms (ECGs). Some degree of ST-segment elevation is common, especially in young men. Commonly referred to as benign early repolarization (BER), this elevation is seen in the precordial leads. Due to the prevalence of this finding, it is not a normal variant, but rather a normal finding. Here is an unusual case of acute myocardial infarction (MI) presenting as BER. In this case, the ECG did not show typical ST elevation or evolve over time, showing changes of Q waves as expected with ST-segment elevation myocardial infarction. However, the point-of-care troponin was elevated, and the patient was then diagnosed as having acute MI.

## Introduction

Benign early repolarization (BER), as its name suggests, is a benign electrocardiogram (ECG) pattern usually seen in young individuals; particularly, this is an ST-segment elevation that is not considered pathological. Rosen's Emergency Medicine [1] mentions that both benign and pathologic ST-segment elevation are frequent ECG findings in adults presenting with chest pain. In young men, it is commonly observed in up to 90% of cases. This elevation, commonly called BER, is primarily visible in the precordial or chest leads. The ST-segment elevation of BER is typically elevation of more than or equal to 1 mm in men, while it is usually less than or equal to 1 mm in women. The elevation is similar to that seen with an ST-segment elevation myocardial infarction (STEMI); it is concave and becomes more appreciable as the following S wave (the negative deflection of the QRS complex) becomes deeper. One key factor that distinguishes between benign ST elevation and the pathologic elevation seen in STEMI is that STEMI is a dynamic phenomenon. Therefore, ECGs taken over time during fluctuating symptoms should show some variation in the degree of ST-segment deviation in the presence of acute coronary syndrome [2]. This benign ECG pattern is characterized by widespread ST-segment elevation, commonly seen in young, healthy patients under the age of 50. This phenomenon is also sometimes referred to as "high take off" or "J-point elevation," which can also mimic pericarditis apart from acute myocardial infarction (MI) [1]. A particular way of identifying and differentiating one from the other is that in BER, reciprocal ST-segment depression is only seen in lead augmented vector right. The presence of ST-segment depression in several other contiguous leads strongly suggests the diagnosis of STEMI [3].

In this case study, we look at a young patient presenting with nonspecific symptoms and an ECG suggestive of changes of BER, yet was later diagnosed with an underlying life-threatening cardiac issue, which could have easily been missed.

## Case presentation

In this case, we have a 25-year-old man who presented to the emergency department (ED) with a burning sensation in his upper abdomen, which is radiating to his chest and left shoulder. These symptoms had persisted overnight. The patient had no significant medical history and worked as a delivery boy at a local restaurant. He confirmed that he was not on any medication and denied using illicit substances. There were no prior reports of chest pain during exercise or at rest. His family history was also unremarkable, with no occurrences of familial hyperlipidemia or sudden death.

**Figure 1:**
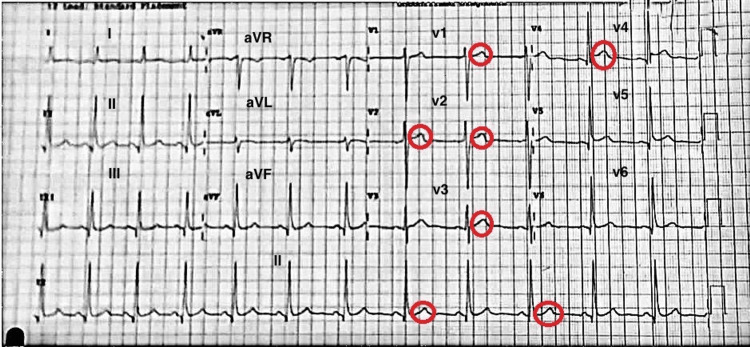
The initial electrocardiogram showed a normal sinus rhythm with an early repolarization pattern in the V4 and V5 leads with J-point elevation aVR: augmented vector right; aVL: augmented vector left; aVF: augmented vector foot

On physical examination, the patient weighed 80 kg and was 178 cm in height. His vital signs were as follows: SpO_2_ 99%, heart rate 61 bpm, respiratory rate 18, and BP 138/84 mmHg. He appeared uncomfortable, without sweating or fever, but presented with mild epigastric tenderness. An ECG revealed a normal sinus rhythm with an early repolarization pattern in leads V3, V4, V5, and V6, displaying J-point and ST-segment elevations (Figure 1). No reciprocal ST-segment depression was observed. Initially, this was considered a case of BER in a low-risk young man with a low Heart Score (a Heart Score includes history, ECG changes, age, presence of risk factors, and initial troponin).

However, the point-of-care (POC) troponin levels requested for the initial screening came back unexpectedly above baseline and were noted to be 26 ng/mL (0.00-0.04 ng/mL). Due to this rising concern of an acute myocardial injury or MI, the treatment plan was adjusted.

Full blood tests, including renal function tests, were sent to the lab, and the CXR was noted to be unremarkable. Despite the lack of a cardiac history, to rule out MI, a repeat ECG was performed, but no changes from the initial ECG were observed, even though the POC troponin remained elevated (repeat troponin levels of 28 ng/mL at four hours). The cardiology team was consulted to determine and investigate the possibility that the patient might have had an MI. A bedside echocardiogram was performed, and it revealed an anterior wall motion abnormality. Hence, the patient was urgently transferred to the catheterization lab for diagnostic coronary angiography and, subsequently, if necessary, a percutaneous coronary intervention.

**Figure 2:**
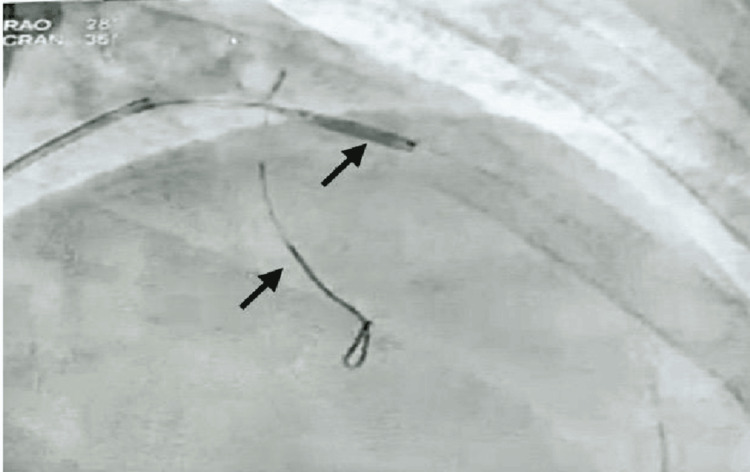
Coronary angiography with a near total occlusion of the LAD coronary artery can be seen here, as no contrast is visible in the LAD artery secondary to occlusion (coronary vasculature not visible at the end of the guide wire) LAD: left anterior descending

**Figure 3:**
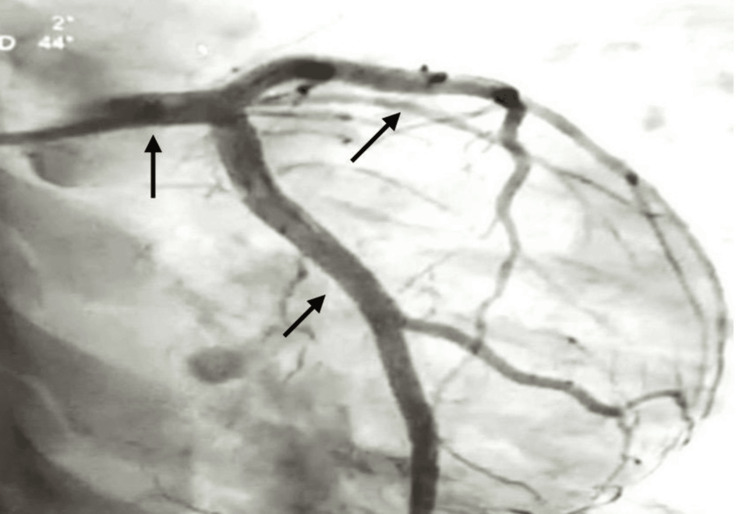
Coronary angiography image after further exploration of the occlusion site on LAD, providing more detailed visualization of the coronary anatomy, as the contrast media can be seen passing through the vasculature (the detailed vasculature is now visible after manipulation of the occlusion and stent insertion) LAD: left anterior descending

The coronary angiography revealed almost total occlusion of the left anterior descending (LAD) coronary artery, which was not expected, as the patient was young, with a low HEART score, and was not having extensive symptoms, with an ECG showing BER. The only pertinent positive finding raising a concern was the POC troponin levels, which were elevated. Figure 2 shows an ostioproximal near-total occlusion of the LAD coronary artery. This was treated with a drug-eluting stent placement, and normal coronary circulation was achieved, as shown in Figure 3.

The patient was later observed and noted to have improvement in his symptoms and normalization of the cardiac biomarkers (troponin <0.04 ng/mL). The patient was later discharged.

## Discussion

BER is not always benign. Although it is considered a normal variant ECG in young individuals, BER could be a mimic of ST-segment elevation and, therefore, could mask a life-threatening condition underneath. This, in turn, can delay further management of the underlying serious condition.

It is uncommon to see a BER pattern on an ECG in patients older than 50 years of age, especially in those older than 70, where elevation of the ST segment is more likely to indicate myocardial ischemia. The ECG findings for hyperacute anteroseptal STEMI typically include ST-segment elevation and hyperacute T waves in leads V2-V4, as well as ST elevation in leads I and augmented vector left. Additionally, there may be appropriate ST depression in lead III and the presence of Q waves in the septal leads (V1 and V2). In contrast, the ECG findings associated with BER may show a 1 mm elevation of the ST segment, a T wave height of 6 mm, resulting in an ST/T wave ratio of 0.16. An ST/T wave ratio of <0.25 is indicative of BER. ST elevation of multiple leads, similar to acute MI or pericarditis, can also be seen in BER. Differentiating between these two can be challenging, especially in young patients. Moreover, this ECG pattern could be found in up to 10%-15% of patients presenting at the ED with chest pain. This makes it a common diagnostic dilemma. While this pattern was initially introduced as a benign ECG pattern, further studies have shown connections between this BER and sudden cardiac death (SCD). A study in 2008 found that the incidence of BER patterns was higher in patients who were resuscitated after developing cardiac arrest due to idiopathic ventricular fibrillation (VF) (odds ratio, 10.9), with these patterns identified on premorbid ECGs [4]. A 2009 study in a Finnish cohort found that an increased risk of cardiac death was associated with an early repolarization pattern in the inferior leads in middle-aged patients over a 30-year follow-up [5]. This was published in the New England Journal of Medicine. A similar pattern present after a successful resuscitation event is known as early repolarization syndrome (ERS). This refers to a BER pattern on ECG in patients who have been successfully resuscitated after a cardiac arrest due to VF of an idiopathic nature. As suggested in a study conducted earlier on BER, the onset or recurrence of idiopathic VF may be predisposed by global early repolarization rather than repolarization changes in isolated lateral leads [2]. Another study suggested a connection between SCD and the presence of ERS, especially when there is a family history of syncope [6]. A study of long-term prognosis of the early repolarization pattern in a group with atherosclerotic risk factors showed that the early repolarization pattern was associated with an absolute risk increase of 52.3 additional SCDs per 100,000 person-years in the population at high risk for atherosclerotic heart disease [7]. While these patterns are difficult to distinguish, studies have been done to find any identifiable differences to help make a diagnosis. One such retrospective study with 355 cases of anterior STEMIs over two years showed that while 143 had subtle changes, the other 171 were typical anterior STEMI; changes were compared with early repolarization ECGs. This suggested some differentiating factors between subtle anterior STEMI and early repolarization through the means of ECGs. In STEMI, the ST-segment elevation was more significant, the R-wave amplitude was lower, and the ratio of T-wave amplitude to R-wave amplitude was higher in leads V2, V3, and V4 compared to early repolarization [8].

In our case, the patient presented with chest pain with no typical findings of STEMI on the ECG, but rather a BER pattern. There were no dynamic changes seen over time, and the diagnosis was guided by troponin levels. This is uncommon to see even with a BER pattern, as the dynamic changes would still be expected with an evolving MI. This makes differentiating even further difficult. Nonetheless, the serial ECGs should be reviewed when such a diagnostic dilemma arises and should be correlated with changes in troponin levels and symptoms at presentation. Even though current literature suggests there are some differentiating factors, exceptions such as these can still occur, which requires an even deeper degree of concern while reviewing a young patient with chest pain and an ECG that does not correlate.

As important as it is to identify a serious disease and treat it, it is equally important not to overtreat a benign condition. As mentioned in earlier parts, the presence of BER in a young individual is considered a normal variant; hence, risk factors also need to be weighed while making a decision. Simple bedside procedures and skills can be useful in these situations, such as a bedside Echo to rule out wall motion abnormalities, and if in doubt, a specialist should be contacted.

## Conclusions

BER, though often considered a normal ECG variant in young individuals, can mimic STEMI and, by doing so, can potentially lead to diagnostic confusion with serious underlying conditions, leading to delayed diagnosis and management. While BER is typically benign, in certain cases, especially in younger patients with chest pain and early repolarization patterns on ECG, it may signal an increased risk for adverse cardiac events. Clinicians should maintain a high index of suspicion when evaluating patients. They need to carefully assess the features of the ECG and correlate these findings with the patient's presenting complaints, levels of cardiac markers, and family history. This evaluation is essential for distinguishing BER from more serious conditions such as STEMI or pericarditis, even in patients who do not exhibit traditional cardiovascular risk factors.
